# Curcumin-Mediated HDAC Inhibition Suppresses the DNA Damage Response and Contributes to Increased DNA Damage Sensitivity

**DOI:** 10.1371/journal.pone.0134110

**Published:** 2015-07-28

**Authors:** Shu-Huei Wang, Pei-Ya Lin, Ya-Chen Chiu, Ju-Sui Huang, Yi-Tsen Kuo, Jen-Chine Wu, Chin-Chuan Chen

**Affiliations:** 1 Department of Anatomy and Cell Biology, College of Medicine, National Taiwan University, Taipei, Taiwan; 2 Graduate Institute of Natural Products, Chang Gung University, Taoyuan, Taiwan; 3 Institute of Stem Cell and Translational Cancer Research, Chang Gung Memorial Hospital, Taoyuan, Taiwan; 4 Chinese Herbal Medicine Research Team, Healthy Aging Research Center, Chang Gung University, Taoyuan, Taiwan; The University of Hong Kong, HONG KONG

## Abstract

Chemo- and radiotherapy cause multiple forms of DNA damage and lead to the death of cancer cells. Inhibitors of the DNA damage response are candidate drugs for use in combination therapies to increase the efficacy of such treatments. In this study, we show that curcumin, a plant polyphenol, sensitizes budding yeast to DNA damage by counteracting the DNA damage response. Following DNA damage, the Mec1-dependent DNA damage checkpoint is inactivated and Rad52 recombinase is degraded by curcumin, which results in deficiencies in double-stand break repair. Additive effects on damage-induced apoptosis and the inhibition of damage-induced autophagy by curcumin were observed. Moreover, *rpd3* mutants were found to mimic the curcumin-induced suppression of the DNA damage response. In contrast, *hat1* mutants were resistant to DNA damage, and Rad52 degradation was impaired following curcumin treatment. These results indicate that the histone deacetylase inhibitor activity of curcumin is critical to DSB repair and DNA damage sensitivity.

## Introduction

Double-strand breaks (DSBs) are among the most cytotoxic forms of DNA damage and result in genomic instability if not properly repaired. DSBs can be generated by endogenous cell stress or by exogenous agents, such as chemotherapeutic drugs or ionizing radiation. Intrachromosomal DSBs trigger the DNA damage checkpoint, leading to arrest of the cell cycle to allow time for DNA repair[1]. The DNA damage checkpoint in budding yeast is initiated by two PI3 kinases, Tel1 and Mec1 (ATM and ATR in mammals, respectively)[2]. Mec1 and its binding partner Ddc2 (ATRIP in mammals) then activate multiple targets through phosphorylation, including histone H2A, a key regulatory element in repair protein recruitment. Mec1-Ddc2 also activates Rad9 adaptor protein to mediate the phosphorylation of Rad53 kinase (CHK2 in mammals)[3]. Activation of Rad53 plays an important role in the arrest of the cell cycle at the metaphase-to-anaphase transition[4]. Proper DNA repair is critical to normal cell survival. However, efficient DNA repair processes allow tumor cells to survive DNA damage caused by radio- or chemotherapeutic treatment. Thus, inhibitors of DNA repair pathways may enhance the therapeutic effects of radiotherapy or DNA-damaging chemotherapeutic drugs[5, 6]. Indeed, inhibitors of proteins involved in DNA repair, including ATM, ATR, CHK1, CHK2, DNA-PK, MGMT, and PARP, have been used in combination with chemotherapy and/or radiotherapy[7, 8].

Curcumin, a compound found in the plant *Curcuma longa*, has been shown to sensitize tumor cells to DNA-damaging drugs and ionizing radiation[9, 10], suggesting that curcumin increases DNA damage sensitivity by suppressing the DDR[10]. Curcumin also inhibits histone deacetylase (HDAC) activity in many cell types[11–15]. Because histone deacetylase inhibitors are known to suppress the DDR[16], it is likely that the HDAC activity inhibited by curcumin is important for the suppression of DNA repair. However, the mechanisms behind these activities are largely unknown due to the difficulties associated with studying the repair of sequence-independent DNA damage.

In this study, we used an inducible defined DSB (SSA system)[17] that allowed us to take advantage of the ease of manipulation of budding yeast to study localized DNA damage and the influence of curcumin treatment on DNA repair. Using this system, we found that curcumin sensitizes yeast cells to DNA damage, presumably by inhibiting the G2/M DNA damage checkpoint and promoting Rad52 degradation. In addition to determining the roles of curcumin in the DDR, we also observed the effects of curcumin on damage-induced apoptosis and autophagy. We further showed that the inhibition of Rad52 and DNA repair by curcumin requires its HDAC inhibitor activity. Our results suggest a critical role for DDR in HDAC inhibition-mediated DNA damage sensitivity, which could provide a valuable model for studying the combined use of HDAC inhibitor in radiotherapy and/or chemotherapy.

## Materials and Methods

### Strains, plasmids, and chemicals

All yeast strains are described in S1 Table. Curcumin (≥ 94% curcuminoid content), protease inhibitor cocktail, Annexin V-FITC apoptosis kit, nocodazole, methyl methanesulfonate (MMS), and 4-nitroquinoline-1-oxide (4NQO) were purchased from Sigma-Aldrich. FM 4–64 was purchased from Biotium. Phenylmethanesulfonyl fluoride was purchased from Enzo Life Sciences. Hydroxyurea (HU) was purchased from Santa Cruz Biotechnology. The plasmid expressing the GFP fusion of Atg8 was a gift from Dr. Wei-Pang Huang (National Taiwan University).

### DNA damage sensitivity plate assay

Yeast cells were plated as five-fold serial dilutions onto yeast extract-peptone-dextrose (YPD), yeast extract-peptone (YEP) medium containing 2% galactose or the indicated concentrations of DNA-damaging compound. The cells were allowed to grow at 30°C for 2–3 days and photographed to record colony formation.

### HO induction

Cultures were grown for 12 hours in YEP medium containing lactic acid. Samples were collected for cutting and repair analysis, Rad53 western blots, or ChIP at the time points indicated in the figures; samples at time 0 were taken prior to the addition of galactose (final concentration of 2%).

### Cutting and repair analysis

Cutting and repair of the HO site in the SSA strains was performed using the three primers. (SSA1: CCGCTGAACATACCACGTTG; SSA2: CACTTCCAGATGAGGCGCTG; SSA3: TGAACTCTGGTGTCTTTTAG). PCR amplification prior to repair yields a 1.7-kb product, amplification during DNA damage yields no PCR product, and analysis following repair by SSA yields a 3.0-kb product. Primers corresponding to the *RAD3* gene were included in the multiplex PCR as an internal control.

### Immunoblot analysis

Proteins from mid-log phase cultures were isolated using trichloroacetic acid as described previously[18]. The samples were resolved by electrophoresis on a 10% SDS-polyacrylamide gel. The following primary antibodies were used for immunoblotting at a 1:1000 dilution: Anti-Pgk1 (ab113678), anti-Rad53 (ab104232), and anti γ-H2A (ab15083) were purchased from Abcam; anti-HA (#2578) was purchased from Cell Signaling; anti-Myc (sc-40), anti-Hda1 (sc-6657), anti-Rpd3 (yC-19), and anti-Sir2 (yN-19) were purchased from Santa Cruz Biotechnology; anti-GFP (G1544) was purchased from Sigma-Aldrich; anti-mouse (A9044), anti-rabbit (A0545), and anti-goat (A5420) HRP-linked secondary antibodies were purchased from Sigma-Aldrich and used at a 1:100000 dilution. Images were acquired with a Wealtec KETA-CL imaging system.

### Chromatin immunoprecipitation (ChIP) assay

ChIP assays were performed as previously described[19]. In brief, protein-DNA complexes were cross-linked with formaldehyde followed by shearing of the chromatin and immunoprecipitation. Quantitation of the DNA molecules present in immunoprecipitates was performed with qPCR using a Roche 480 instrument. Anti-HA (Cell Signaling, #2367,), anti-Myc (Santa Cruz Biotechnology, sc-40) and anti-Rfa1 (Agrisera, ab15083) antibodies were used for ChIP (1:50).

### Fluorescence microscopy

Fluorescence microscopy analyses were performed using a Nikon ECLIPSE Ni-U plus fluorescence microscope equipped with 100X oil objectives. Images were acquired with a DS-U3 CCD camera and controlled using NIS-Element BR 4.0 software.

## Results

### Curcumin sensitizes yeast cells to DNA-damaging drugs

**Fig 1:**
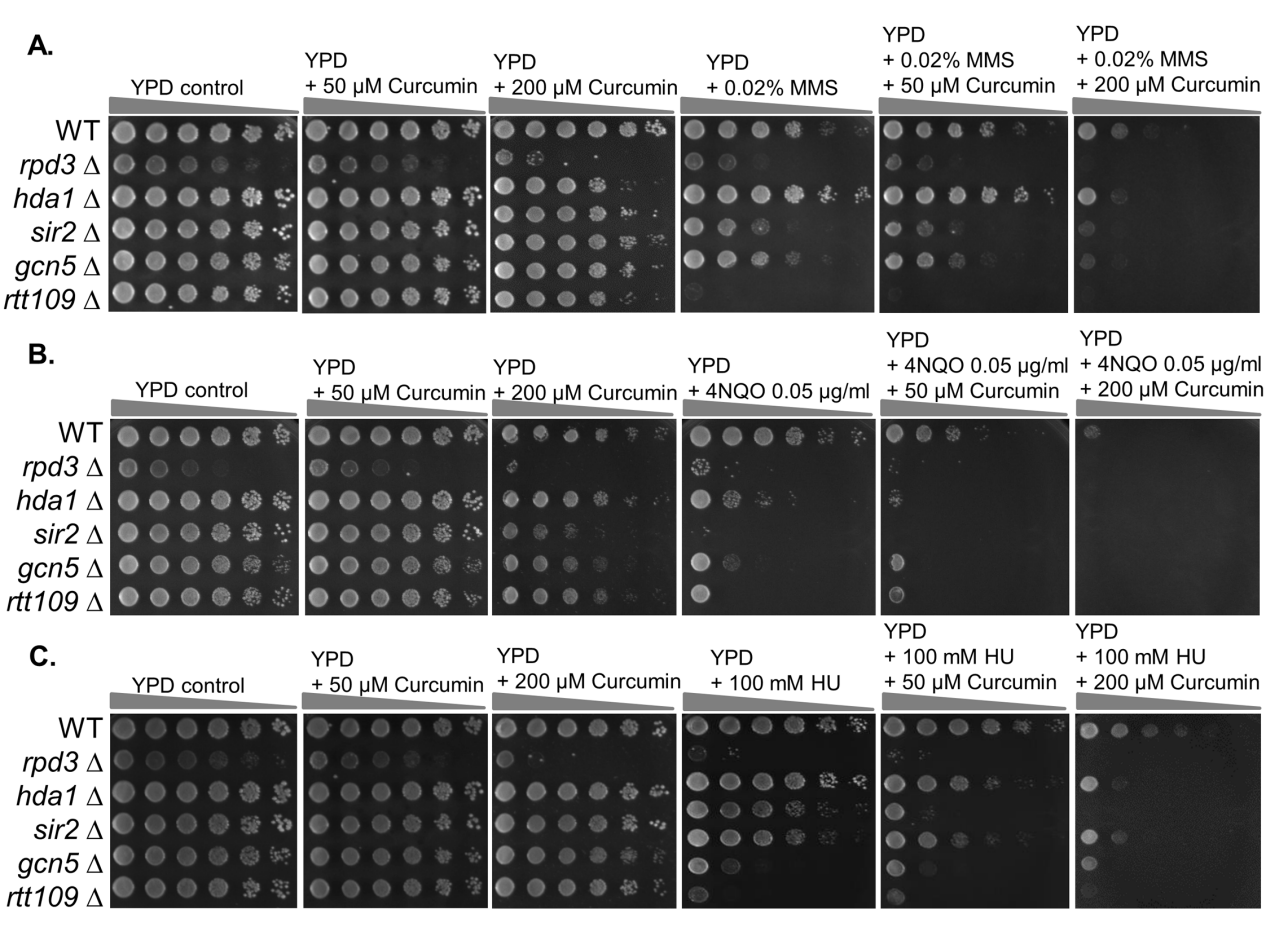
Curcumin sensitizes wild-type cells to the DNA-damaging drugs. Five-fold serial dilution analysis of the indicated isogenic strains, including WT (BY4741), *rpd3∆* (BY4741-rpd3), *hda1∆* (BY4741-hda1), *sir2∆* (BY4741-sir2), *gcn5∆* (BY4741-gcn5), and *rtt109∆* (BY4741-rtt109) shows sensitivity to (A) MMS, (B) 4NQO, and (C) HU with or without curcumin. The cells were allowed to grow at 30°C for 3 days and photographed to record colony formation.

To investigate whether curcumin increases the DNA damage sensitivity in budding yeast following treatment with DNA-damaging drugs, we plated wild-type cells and control strains onto YPD with curcumin and DNA-damaging drugs. HDAC inhibition has been linked to suppression of the DNA damage response [20]. Cells lacking Rtt109 and Gcn5 are hypersensitive to DNA-damaging agents [21, 22]. Indeed, we found that *rpd3* (Class I HDAC), *hda1* (Class I HDAC), *sir2* (Class I HDAC), *rtt109*, and *gcn5* mutants were sensitive to DNA-damaging drugs (Fig 1). We also found that low doses of curcumin or DNA-damaging drugs did not inhibit the growth of wild-type cells (Fig 1). However, wild-type cells became very sensitive to MMS (a DNA alkylating agent), 4NQO (an ultraviolet-mimetic agent) and HU (a DNA replication-dependent damaging drug) when combined with curcumin. The degree of sensitivity depended on the dose of curcumin (Fig 1). This result demonstrates that curcumin sensitizes yeast cells to DNA-damaging drugs. We observed that wild-type cells showed lower sensitivity to the combination of HU (a DNA replication-dependent damaging drug) with curcumin (Fig 1C), suggesting that curcumin may increase the sensitivity of yeast cells to DNA damage in a replication-independent manner.

### Curcumin inhibits DNA double-strand break repair

**Fig 2:**
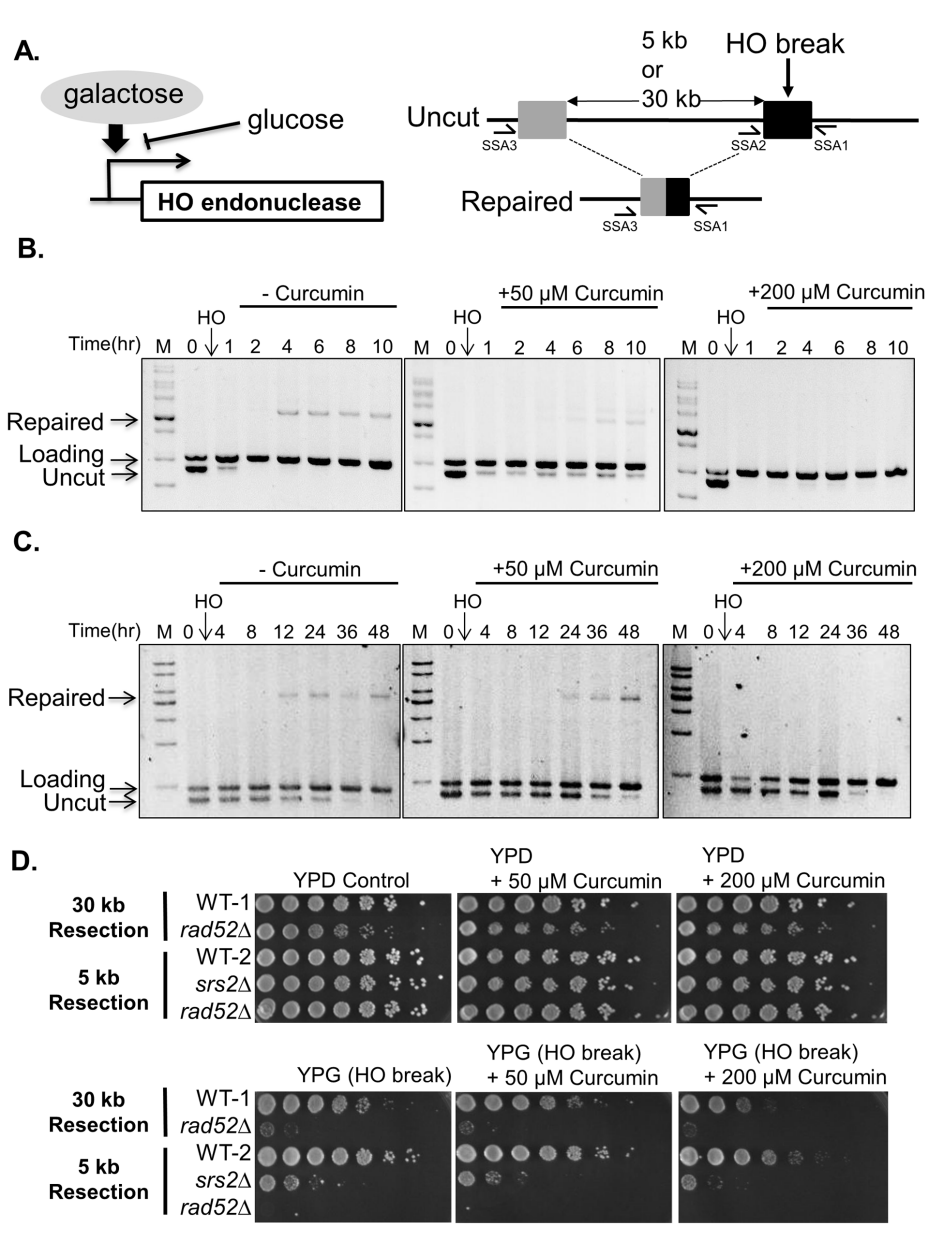
Curcumin increases DNA damage sensitivity and inhibits DNA double-strand break repair in SSA strains. (A) A schematic diagram of the SSA system. Galactose was used to induce HO endonuclease to generate a specific HO lesion. Repair of the HO lesion at the HO cleavage site (black box) requires 5-kb or 30-kb of resection back to the uncleavable HO cleavage site (gray box). Three PCR primers were used to measure the DNA damage and repair. (B) Curcumin inhibits DNA double-strand break repair in the 5-kb resection strains. The repair of the HO lesion was analyzed by PCR. The DSB was induced by the addition of galactose to the 5-kb resection strain (YMV045). After 30 min, cultures were treated with 50 μM and 200 μM curcumin. (C) Curcumin inhibits DNA double-strand break repair in the 30-kb resection strain. PCR analysis of the 30-kb resection strain (YMV002) as described in (B). (D) Five-fold serial dilution analysis of WT-1 (YMV002), *rad52∆* (YMV037), WT-2 (YMV045), *srs2∆* (YMV057), and *rad52∆* (YMV046) shows the sensitivity to galactose and curcumin. The cells were allowed to grow at 30°C for 3 days and photographed to record colony formation.

We wanted to ask whether curcumin sensitizes yeast cells to DNA damage by inhibiting the DNA repair machinery. Because DNA-damaging agents cause damage at random sites, it is difficult to assay DNA repair following treatment with such agents. We used an HO endonuclease-mediated system (SSA system) to induce a single DSB, allowing us to investigate the kinetics of DNA repair and activation of the DNA damage checkpoint (Fig 2A)[17]. The SSA strains contain an uncleavable region (Fig 2, gray box) that is homologous to the HO site (Fig 2, black box) located either 5-kb or 30-kb proximal to the cleavable HO site on chromosome III. Upon induction of the HO break, the 5' ends are resected, exposing the region homologous to the uncleavable HO site. These ends then anneal together, and the intermediate can be processed by digesting away the single-stranded tails. Therefore, the repaired products can be monitored by PCR. Using PCR analysis, we found that curcumin inhibited DSB repair in SSA strains and caused a concentration-dependent decrease in DNA repair in 5-kb and 30-kb SSA strains (Fig 2B and 2C). To confirm that curcumin increases the DNA damage sensitivity in SSA strains similarly to the effects observed following treatment with DNA-damaging drugs, we plated SSA strains onto galactose (YPG) plates to induce an HO lesion. As a control, we included a rad52 mutant that failed to repair the HO lesion[19]. We found that wild-type cells were more sensitive to curcumin and YPG (HO lesion) together than to YPG or curcumin alone (Fig 2D). Furthermore, the degree of sensitivity also depended on the length of resection required in SSA strains. These results demonstrate that curcumin sensitizes SSA strains to a single DSB and suggest that curcumin sensitizes yeast cells to DNA damage by inhibiting DSB repair.

### Curcumin inhibits the G2/M DNA damage checkpoint

**Fig 3:**
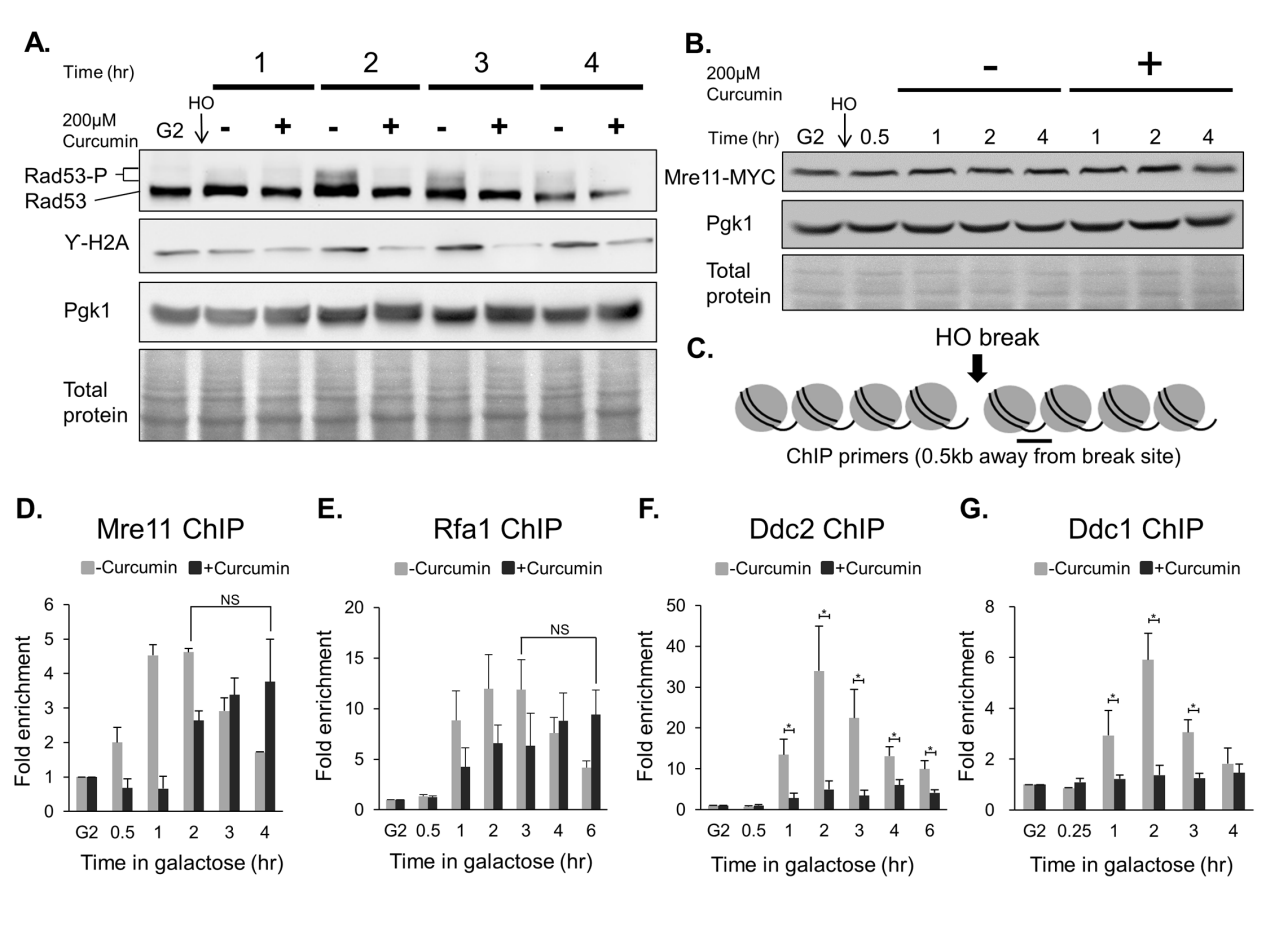
Curcumin influences the DNA damage response. (A) Wild-type cells (YMV045) or (B) *MRE11-MYC* cells (YAY032) were arrested at G2 with 15 μg/ml nocodazole, and HO endonuclease was induced by the addition of galactose to generate a DSB. After 30 min, the cultures were divided equally and treated with or without 200 μM curcumin. The indicated antibodies were used to detect protein expression by western blotting. Amido black staining of total proteins and Pgk1 protein levels serve as loading controls. (C) Schematic of the positions of the primers used for ChIP analyses. (D) *MRE11-MYC* cells (YAY032), (E) *RFA1-MYC* cells (YAY022), (F) wild-type cells (YMV045), or (G) *DDC1-MYC* cells (CCY025) were cultured as in (A), and the recruitment of the indicated proteins flanking the HO lesion was analyzed by ChIP. Error bars represent the standard deviations of at least three independent experiments. Asterisks (*P<0.05) represent a significant difference between curcumin-treated and untreated cells. (n = 3 for (D), (F) and (G). n = 5 for (E)).

Next, we investigated how curcumin affects the DNA repair machinery in yeast. The repair of DSBs usually depends on the DNA damage checkpoint, which arrests cell cycle progression until the damage has been repaired[1]. We tested whether curcumin influences the DNA damage checkpoint following a DSB. After HO induction of DSB during G2, we found that curcumin prevented phosphorylation of Rad53 and H2A (Fig 3A), indicating that curcumin inhibits the G2/M DNA damage checkpoint. Next, we investigated how curcumin influences the G2/M DNA damage checkpoint by analyzing the early events associated with the repair of DSBs. The cascade of events begins with the accumulation of Mre11, which activates Tel1 and in turn initiates DNA resection[23]. We observed that the Mre11 protein level was not affected by curcumin (Fig 3B). We next determined whether curcumin limits the recruitment of Mre11 to DSBs. Chromatin immunoprecipitation (ChIP) analysis of Mre11 levels in the vicinity of the HO lesion indicated that the kinetics of Mre11 recruitment were delayed with curcumin treatment (Fig 3D). We then determined whether curcumin counteracts the DNA damage checkpoint by preventing DNA resection after a DSB. DNA resection generates replication protein A (RPA)-coated single-stranded DNA (RPA-ssDNA); we used ChIP analysis to examine the binding of Rfa1, a subunit of RPA, to ssDNA in the vicinity of the HO lesion with or without curcumin. Similar to the recruitment of Mre11, we observed the delay in the formation of the RPA-ssDNA complex associated with curcumin treatment (Fig 3E). Although the recruitment of Mre11 and Rfa1 were delayed with curcumin treatment, they can still be recruited to the site of break (P = 0.46 and 0.237 respectively), suggesting that the delay of recruitment may not be the major reason for the inhibition of DNA damage checkpoint by curcumin.

After DNA resection, the central checkpoint kinase Mec1-Ddc2 is recruited to an RPA-ssDNA complex that is critical for the phosphorylation of H2A and activation of the checkpoint[24, 25]. We next studied the recruitment of Ddc2 to the HO lesion following curcumin treatment. Using ChIP analysis, we found that curcumin inhibited the recruitment of Ddc2 to DSBs (Fig 3F); importantly, curcumin did not affect the total protein level of Ddc2 (S1 Fig). Accordingly, Ddc1 recruitment is inhibited by curcumin. Taken together, these data indicate that curcumin counteracts the DNA damage checkpoint by inhibiting the Mec1 (ATR)-dependent pathway.

### Curcumin promotes damage-induced apoptosis

**Fig 4:**
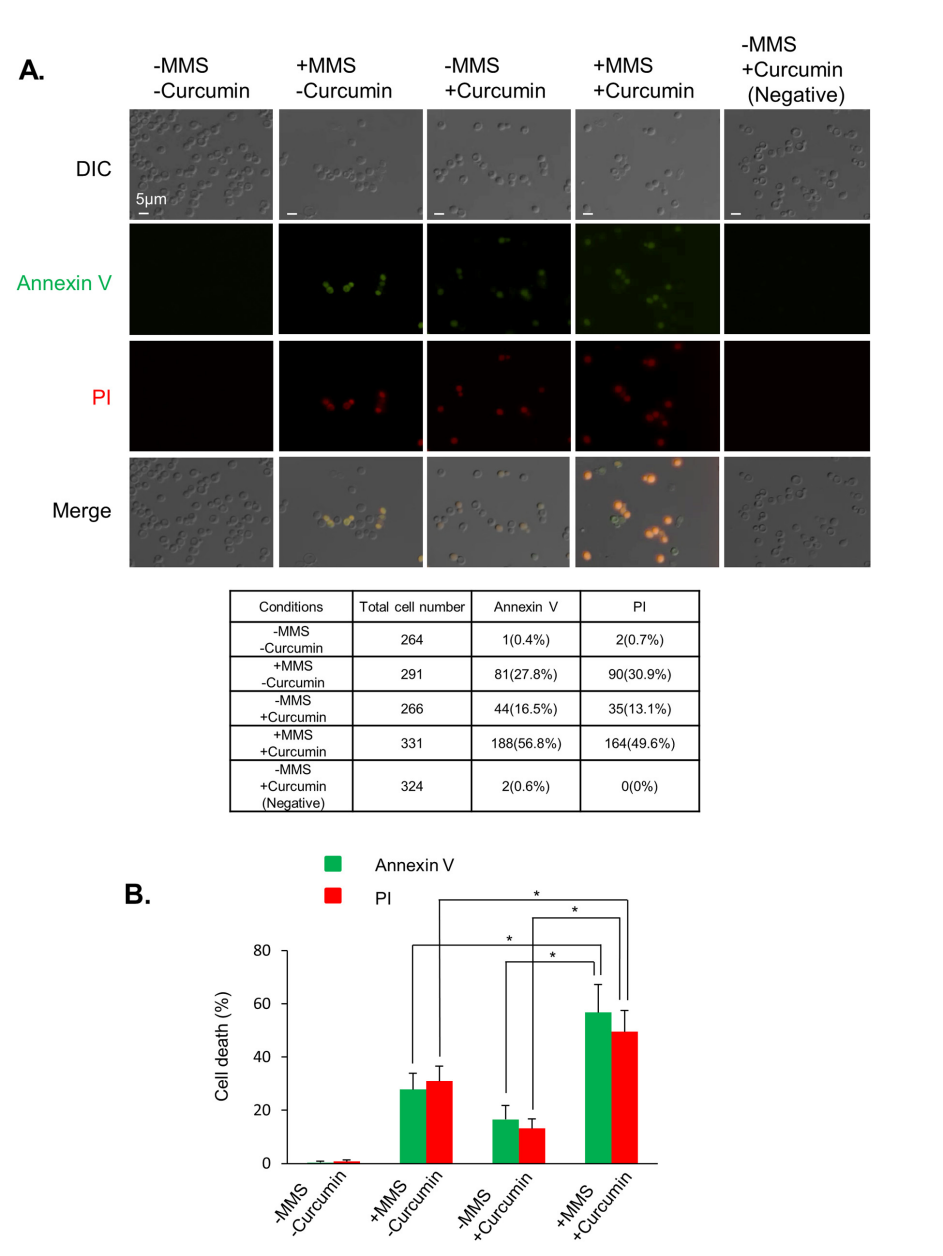
Curcumin cooperatively stimulates apoptosis in response to DNA damage. (A) YMV045 cells were treated with 0.02% MMS for an hour followed by treatment with or without 200 μM curcumin for 3 hours. The fluorescent signals of Annexin V and PI were examined using fluorescence microscopy. Scale bars are 5 μm. The table shows the number of cells expressing Annexin V or PI signals. (B) The panel shows the percentage of fluorescence signals from (A). Error bars represent the standard deviations of three independent experiments. *P<0.05.

We showed that curcumin sensitizes yeast cells to DNA damage. In addition, curcumin counteracts DDR by inhibiting double-strand break repair and inhibiting the G2/M DNA damage checkpoint. This finding led us to investigate whether curcumin-treated cells enter the next cell cycle with unrepaired damage, which cooperatively induces cell death. To test this hypothesis, we examined apoptosis using Annexin V-FITC and PI fluorescence staining. Annexin V- FITC binds to phosphatidylserine and indicates the presence of early apoptosis, while PI stains the late apoptotic and necrotic cells that have compromised membranes. We performed fluorescence staining on yeast cells treated with 0.02% MMS and/or 200 μM curcumin (Fig 4). As curcumin itself may generate a fluorescent background, we included a control with no fluorescent staining (negative). Upon treatment with MMS, 27.8% of the cells showed early apoptotic activity, and 30.9% of the cells showed late apoptotic activity. Consistent with previous reports, curcumin induced apoptosis; 16.5% of the cells showed early apoptotic activity and 13.1% of the cells showed late apoptotic activity[26]. In cells treated with both MMS and curcumin, the percentages of early and late apoptotic cells (56.8% and 49.6%, respectively) generally increased. This result demonstrates that curcumin cooperatively promotes damage-induced apoptosis.

### Curcumin suppresses damage-induced autophagy

**Fig 5:**
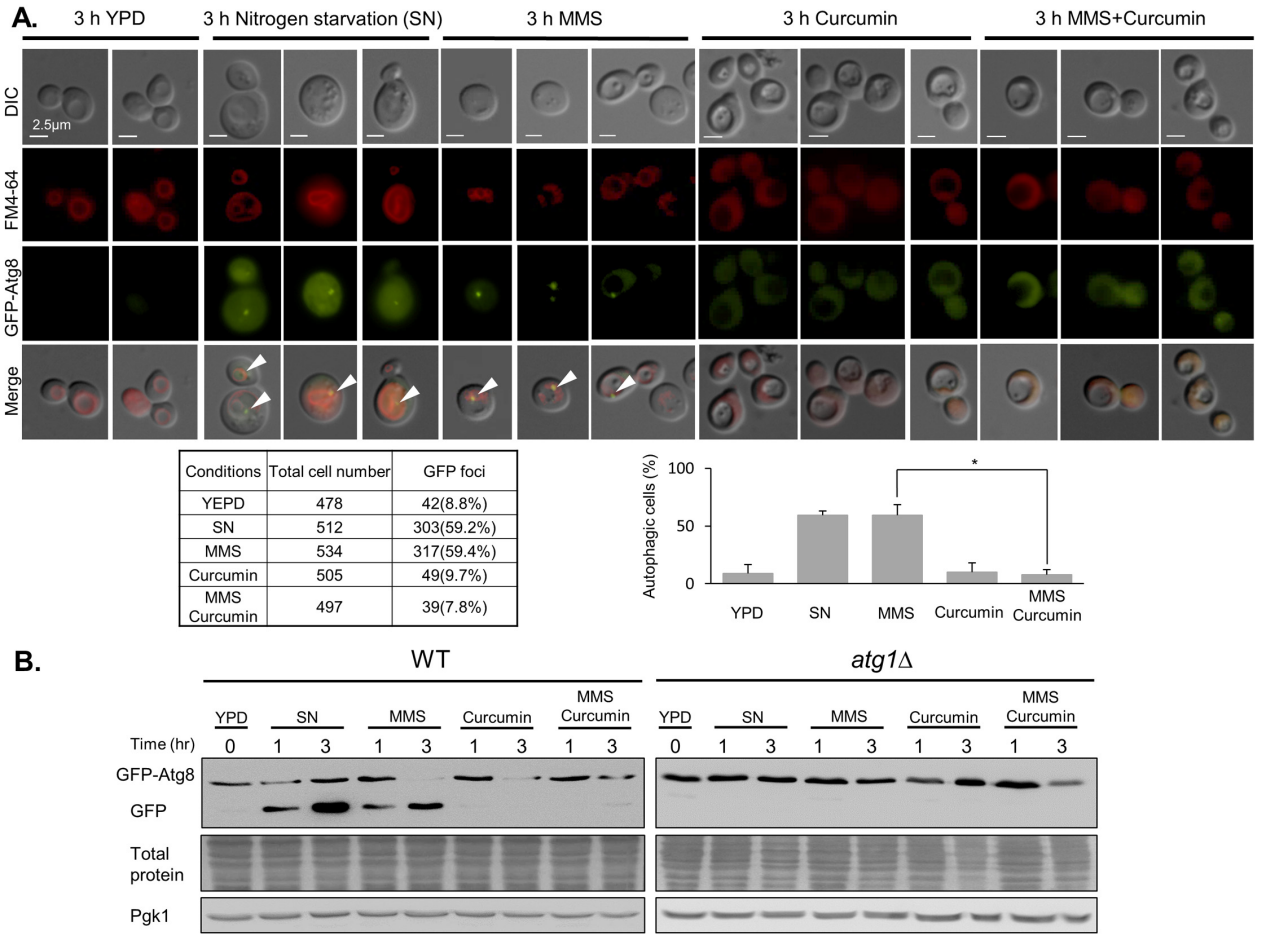
Curcumin inhibits MMS-induced autophagy. (A) The *atg8△* cells were transformed with a plasmid containing a GFP fusion of *ATG8* (RLY004). The RLY004 cells were treated with 0.02% MMS, 200 μM curcumin, or both for 3 hours and processed for fluorescence microscopy. Arrows indicate the accumulation of GFP-Atg8 in the vacuole. FM 4–64 was used to label the vacuoles. Cells cultured in nitrogen starvation medium (SN) served as positive controls for autophagy. Scale bars are 2.5 μm. The table shows the number of cells expressing GFP foci. The panel shows the percentage of fluorescence signals. Error bars represent the standard deviations of three independent experiments. *P<0.05. (B) As in (A), cells were subjected to immunoblotting analysis using anti-GFP antibodies. The *atg1△* cells expressing GFP-Atg8 (RLY005) provide negative controls for autophagy. Amido black staining of total proteins and Pgk1 protein levels serve as loading controls.

Autophagy has been shown to contribute to cell survival. The importance of autophagy in response to DNA damage has recently been reviewed[27]. We determined whether curcumin regulates autophagy following DNA damage. Atg8 is a ubiquitin-like protein that accumulates in the vacuole during autophagy[28]. GFP-tagged Atg8 (GFP-Atg8) is used to detect its delivery to the vacuole by fluorescence microscopy. Fluorescence microscopy analysis revealed the induction of autophagy under conditions of nitrogen starvation as a positive control. MMS-treated cells showed accumulated GFP-Atg8 at levels similar to those of cells cultured under nitrogen starvation (Fig 5A). Specifically, cells treated with MMS and curcumin had few GFP-Atg8 foci. This result demonstrates that curcumin inhibits MMS-induced autophagy. GFP-Atg8 is degraded after delivery into the vacuole, whereas the free form of GFP is relatively stable[29]. Thus, the appearance of free GFP was also used to detect autophagy by immunoblotting. Consistent with these results, cells treated with MMS showed increased levels of free GFP, whereas cells treated with both MMS and curcumin did not undergo the separation of GFP from GFP-Atg8 (Fig 5B). Similar experiments were performed in *atg1* mutants, which are defective in autophagy. Taken together, these data indicate that curcumin suppresses damage-induced autophagy.

### Curcumin inhibits the expression of Rad52 recombinase following DSB

**Fig 6:**
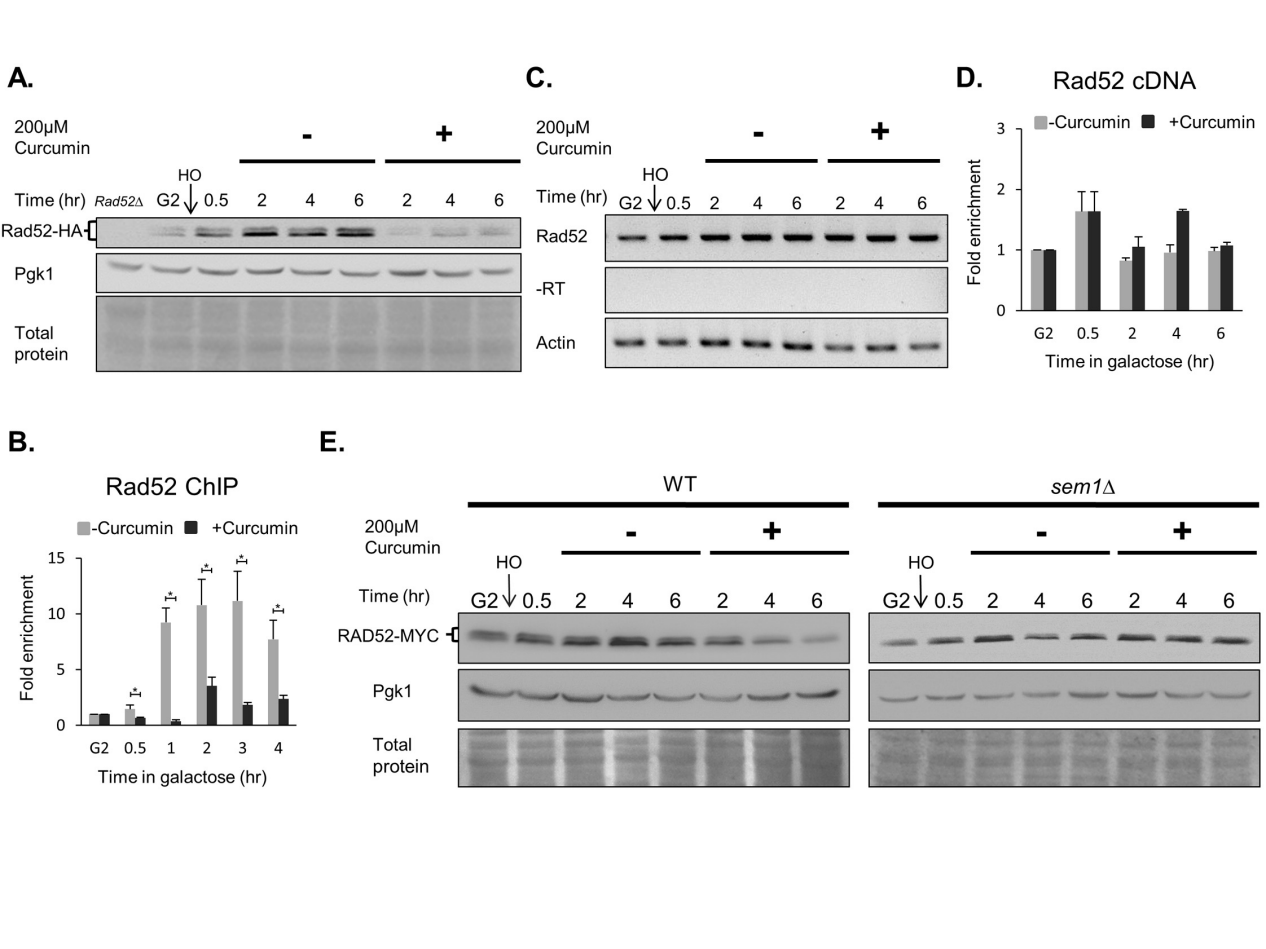
Rad52 protein expression was inhibited by curcumin following DNA damage. *RAD52-HA* cells (YAY013) were arrested in G2 with nocodazole, and HO endonuclease was induced by the addition of galactose to generate a DSB. After 30 min, the cultures were divided equally and treated with or without 200 μM curcumin. (A) Immunoblotting analyses of Rad52 using HA antibodies at the indicated time points. (B) The recruitment of Rad52 to DSBs was analyzed by ChIP. Error bars represent the standard deviations of three independent experiments. *P<0.05. (C) Samples were processed for reverse transcription to generate cDNA and analyzed by PCR or (D) quantitative PCR. Error bars represent the standard deviations of three independent experiments. (E) The deletion of *SEM1* counteracts the disappearance of Rad52 after curcumin treatment following an HO-induced break. Immunoblotting analysis of the *RAD52-MYC* (YAY028) and *sem1△ RAD52-MYC* (RLY006) strains was performed as described in Fig 3A. Amido black staining of total proteins and Pgk1 protein levels serve as loading controls.

Homologous recombination is the next major step in the DNA repair pathway after DNA end resection. To test whether the recombinase is affected by curcumin in response to DSB, we first analyzed the levels of recombinases and found that the levels of Rad52 are reduced by curcumin following DSB (Fig 6A). As a consequence, the recruitment of Rad52 is inhibited by curcumin following DSB (Fig 6B). To determine whether curcumin inhibits Rad52 at the transcriptional level, we examined changes in the Rad52 mRNA levels after treatment with curcumin. Using RT-PCR and RT-QPCR analyses, we found that the Rad52 mRNA levels were similar with or without curcumin treatment (Fig 6C and 6D). Thus, curcumin does not influence Rad52 mRNA expression. To test whether proteasome-mediated degradation of Rad52 is induced by curcumin, we generated a strain deleted for *SEM1*, whose gene product is a 26S proteasome regulatory subunit. We found that yeast lacking *SEM1* restored Rad52 protein levels following treatment with curcumin (Fig 6E). These results reveal that curcumin decreases Rad52 protein levels through proteasome-mediated proteolysis. Because *rad52* mutants fail to repair DSBs[19], these results also suggest that curcumin inhibits DSB repair by inhibiting the expression of Rad52.

### Curcumin inhibits Rad52 and DNA repair in an acetylation-dependent manner

**Fig 7:**
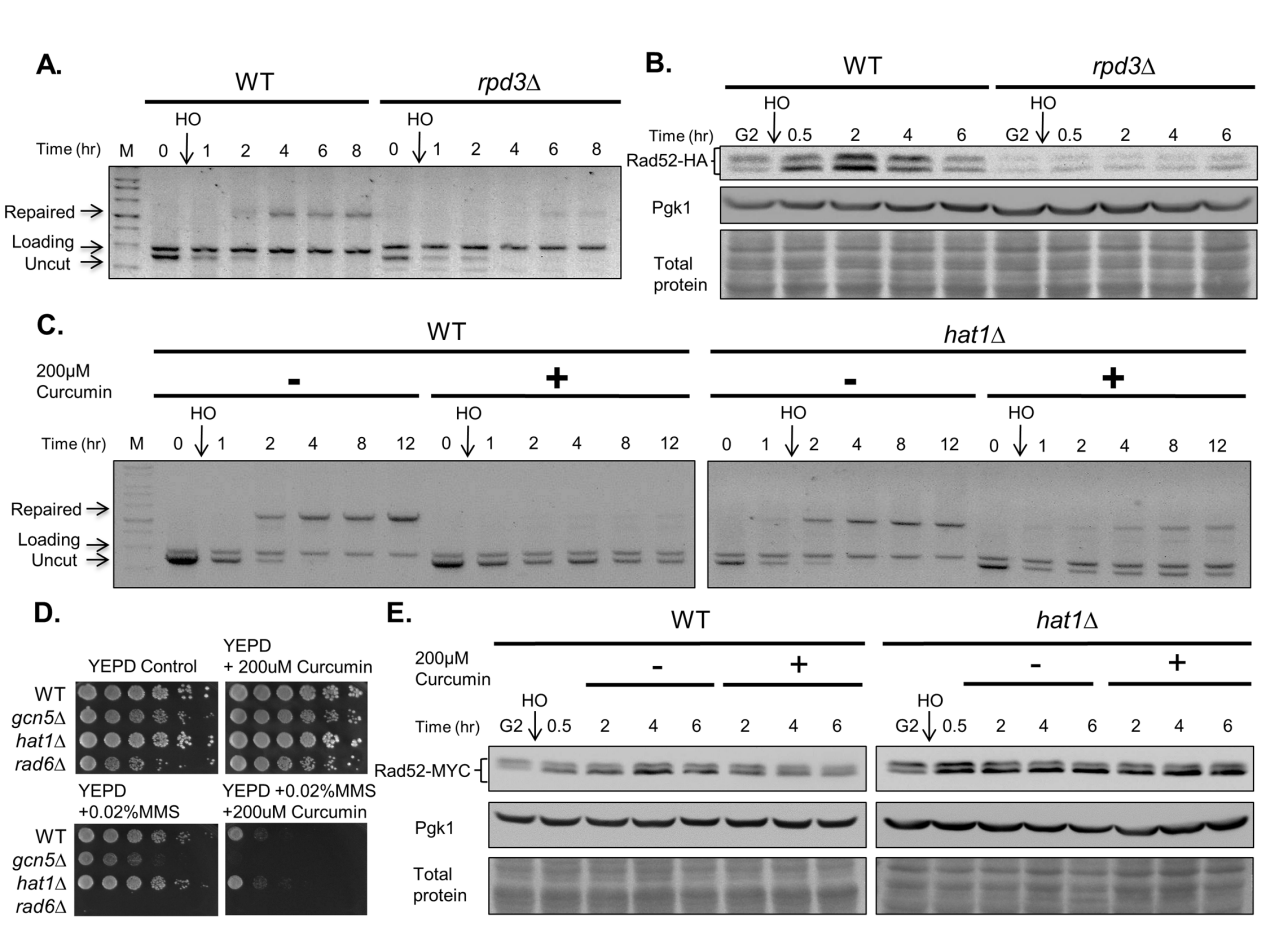
Curcumin inhibits DNA repair processing in an acetylation dependent manner. (A) DNA repair is inhibited in *rpd3* mutants in response to DNA damage. The same analysis presented in Fig 2B was performed on WT (YMV045) and *rpd3∆* (YAY012) strains undergoing SSA with 5 kb resection. (B) The *rpd3* mutants mimic curcumin treatment in response to DNA damage. The *RAD52-HA* (YAY013) and *rpd3∆ RAD52-HA* (YAY016) cells were cultured as in Fig 3A and processed for immunoblotting analysis using HA antibodies. (C) Curcumin failed to inhibit DSB repair in *hat1* mutants. The WT (YMV045) and *hat1∆* (ILY001) strains were used for the repair assay. (D) Five-fold serial dilution analysis of WT (BY4741), *hat1∆* (BY4741-hat1), and *rad6∆* (BY4741-rad6) was performed as described in Fig 1A. (E) The disappearance of Rad52 induced by curcumin is counteracted in *hat1* mutants. Immunoblotting analysis of the *RAD52-MYC* (YAY028) and *hat1△ RAD52-MYC* (RLY007) strains was performed as described in Fig 3A. Amido black staining of total proteins and Pgk1 protein levels serve as loading controls.

Curcumin has been shown to be a more effective HDAC inhibitor than other well-known HDAC inhibitors such as valproic acid[11]. Class I HDAC are negatively affected by curcumin in lymphoma cells[14]. HDAC inhibitors also influence DDR in an acetylation-dependent manner[16]. Therefore, we investigated whether curcumin inhibits Rad52 and DNA repair through its HDAC inhibitor activity. We first tested whether Class I–deficient *rpd3* mutant yeast can mimic curcumin-induced phenotypes. Consist with previous reports, the *rpd3* mutants were hypersensitive to DNA-damaging drugs (Fig 1), and DSB repair was reduced in *rpd3* mutants (Fig 7A)[30, 31]. Moreover, we found that the Rad52 protein levels were reduced in response to DNA damage (Fig 7B). Thus, the inhibition of Rad52 recombinase and DSB repair by curcumin can be mimicked in *rpd3* mutants. The deletion of *RPD3* increases the levels of acetylation of histone and non-histone proteins[16, 32], and it can be counteracted by deleting the histone acetyltransferases (HAT) *GCN5* or *HAT1*[33, 34]. Therefore, we investigated whether the inhibition of DSB repair by curcumin is counteracted in *gcn5* or *hat1* mutants. Similar to its effects in wild-type cells, curcumin inhibited DSB repair and Rad52 stabilization in *gcn5* mutants (S2 Fig). The *gcn5* mutants were sensitive to the combination of curcumin and MMS (Fig 7D). By contrast, curcumin failed to inhibit DSB repair in *hat1* mutants (Fig 7C). Furthermore, curcumin-induced destabilization of Rad52 was attenuated in *hat1* mutants (Fig 7E). The *hat1* mutants were less sensitive to the combination of curcumin and MMS compared to wild-type cells (Fig 7D). These data indicate that Rpd3 and Hat1 control Rad52 degradation in response to DSB. Taken together, these observations suggest that curcumin inhibits DNA repair processing via its HDAC inhibitor activity.

## Discussion

Several reports have demonstrated that curcumin is a potent chemo- and radiosensitizer in many types of cancer[9, 10, 35]. Increasing evidence suggests that curcumin sensitizes tumor cells to different chemotherapeutic agents and to radiation by increasing the sensitivity to DNA damage[36, 37]. However, because of the random generation of DNA damage, the mechanism underlying this effect of curcumin remains elusive. In this study, we used the SSA system, in which a DSB can be generated at a specific site, and we have discovered that curcumin inhibits DDR. Treatment with curcumin selectively increased the sensitivity of yeast cells to DNA-damaging agents but did not affect cells that were not treated with these agents. This result indicates that curcumin may have potential antitumor activity when combined with radiotherapy or chemotherapy. Using the SSA system, we found that curcumin inhibits DSB repair. Thus, it is plausible that curcumin may increase the DNA damage sensitivity by inhibiting DSB repair, as curcumin also sensitized SSA cells to DSBs. The repair of DSBs depends on the activation of the DNA damage checkpoint as well as the regulation of key DNA repair proteins. Activation of the DNA damage checkpoint allows cells to arrest at G2/M for DNA repair. Suppression of checkpoint activation by curcumin is consistent with a previous study showing that curcumin impairs the activation of ATR pathways in mammalian cells[10]. Moreover, we specifically found that curcumin counteracted DNA checkpoint activation by inhibiting the recruitment of Ddc2 and Ddc1, sensors that activate DNA damage checkpoints[38]. We noticed that curcumin slightly inhibits HO cutting. It is unlikely that DDR inhibition is due to the decreased HO cutting by curcumin because the Mre11 protein levels and recruitment are not affected by curcumin.

DNA end resection is critical for DSB repair following activation of the DNA damage checkpoint. Other studies have found that curcumin may inhibit DNA resection, as RPA32 phosphorylation is impaired following DNA damage[10]. By contrast, our data reveal that the formation of the RPA-ssDNA complex is not affected by curcumin. In a previous study, the authors pretreated cells with curcumin, and their results may reflect the protection from DNA damage[39, 40]. By contrast, in our experiment, curcumin was added very shortly after the damage occurred. After the formation of ssDNA, homologous recombination is the next critical process for DSB repair. A significant decrease in the levels of Rad51 and Rad52 recombinant proteins was observed after curcumin treatment following DNA damage, indicating that homologous recombination was inhibited. The inhibition of Rad51 recombinase by curcumin is consistent with previous reports showing that curcumin decreases human RAD51 protein levels following treatment with DNA-damaging drugs in cancer cell lines (S3A Fig)[10, 35]. While Rad51 recombinase is not required for SSA repair in yeast[41], it is unclear whether Rad51 inhibition is essential for increasing the DNA damage sensitivity and, if so, to what degree.

We did not test whether curcumin inhibits non-homologous end joining (NHEJ) because DSBs are mainly repaired by homologous recombination in yeast. Strikingly, the counteraction of DDR by curcumin was observed following galactose-induced damage (SSA system) and following exposure to DNA-damaging drugs (S3B Fig), suggesting that curcumin increased the DNA damage sensitivity following the use of a wide range of DNA-damaging treatments. We propose that when cells pass a DNA damage checkpoint without repairing their DNA, cell death may be exacerbated. Supporting this hypothesis, our findings reveal that curcumin acts cooperatively to induce apoptosis when combined with DNA-damaging drugs. Autophagy is critical not only for cell survival but also for the protection of organisms from stresses[42]. Thus, autophagy is an adaptive response that leads to resistance to therapy-induced apoptosis[43–45]. Although curcumin is reported to induce autophagy in many cell types[43], we found that curcumin impairs DNA damage-induced autophagy, suggesting that curcumin could also circumvent the development of chemotherapy and/or radiotherapy resistance. Further work is required to determine how curcumin contributes to the inhibition of autophagy induced by DNA damage.

Our results reveal for the first time that curcumin decreases the expression of Rad52 that has been up-regulated in response to DSB. Whether the overexpression of Rad52 counteracts the effects of curcumin in the DDR cannot be confirmed, as the overexpression of Rad52 generates DNA repair defects in yeast and mammalian cells[46, 47]. Given that Rad52 is required for DSB repair and *rad52* mutants are hypersensitive to ionizing radiation and DNA-damaging drugs in yeast[48], it is likely that curcumin sensitizes yeast cells to DNA damage by inhibiting the stability of Rad52. In many organisms, homologous recombination is one of the major repair pathways used to repair DSBs. Rad52 recombinase is the only protein that is essential for homologous recombination, and it contributes to many stages of repair, including strand invasion, in *Saccharomyces cerevisiae*[41]. Although human RAD52 is structurally and biochemically similar to yeast Rad52, human RAD52 is largely ignored because depletion of human RAD52 does not results in any obvious defect in homologous recombination[49]. Subsequent findings revealed that RAD52 is functionally redundant with BRCA1/2 in homologous recombination[50]. Moreover, RAD52 reduction is synthetically lethal in combination with reduction of BRCA1/2[51, 52]. Thus, targeting RAD52 in BRCA-deficient tumors might not only enhance the antitumor effect of existing chemotherapeutic drugs but also function as a synthetically lethal therapeutic approach[53, 54].

Our results demonstrate that *rpd3* mutants mimic curcumin-induced suppression of the DNA damage response. Furthermore, *hat1* mutants are resistant to DNA damage, and Rad52 degradation is impaired following curcumin treatment. Therefore, curcumin-mediated HDAC inhibition has been proposed to suppress DDR and contribute to increased DNA damage sensitivity. Consistent with this hypothesis, *rpd3*, *hda1*, and *sir2* mutants are sensitive to DNA-damaging agents. Moreover, we found that curcumin sensitized these HDAC mutants to DNA-damaging drugs, indicating that the HDAC activity inhibited by curcumin is important but not the only mechanism for suppression of DNA repair. The total protein levels of Class I HDACs are decreased by curcumin in Raji cells[14]. However, the Rpd3 Class I HDAC in yeast is not affected by curcumin treatment (S4 Fig). How curcumin regulates Rpd3 activity is unclear. Curcumin post-translationally controls Rad52 protein levels following DNA damage. Because HAT and HDAC control the acetylation levels of histone and non-histone proteins[55], it was tempting to speculate that Rad52 is acetylated and degraded through acetylation-mediated proteasomal degradation. However, the acetylation levels of Rad52 were unchanged following DNA damage and curcumin treatment (data not shown). Hence, the Rad52 protein levels are not directly controlled by acetylation. We also observed that the basal protein level of Rad52 is increased in the absence of Hat1, suggesting that Hat1 may regulate the stabilization of Rad52. The degradation of Rad52 is controlled by sumoylation and affects the efficiency of DNA repair[56], but the sumoylation of Rad52 is not affected by curcumin (data not shown). Although the regulation of Rad52 requires further investigation, we provide a possible model by which HDAC may contribute to the stability of Rad52.

Roberts at al. reported that valproic acid (VPA), an HDAC inhibitor, triggers Sae2 degradation to suppress DNA end resection by increasing the acetylation of Sae2. Here we showed that curcumin suppresses DDR by a distinct mechanism that involves induction of Rad52 degradation to inhibit HR in an acetylation-dependent manner through its HDAC inhibitor activity. DNA end resection is the first step in recombination. Although they have different targets, both VPA and curcumin effectively inhibit HR. Therefore, these drugs may prove to be a potent combination for DNA-damage–based cancer therapy. In contrast to VPA, curcumin is not only an HDAC inhibitor but also a potent HAT inhibitor [57]. Curcumin inhibits Rtt109 (p300/CBP in mammals) activity in yeast and in mammals [58, 59]. HAT mutants (*rtt109* and *gcn5* mutants) are hypersensitive to MMS and 4NQO (Fig 1), suggesting that curcumin may also suppress DDR by its HAT inhibitor activity. However, we saw no SSA repair defect in the absence of Gcn5 (S3 Fig). We also previously observed that rtt109 mutants repair DSB in the SSA system [19], indicating that the inhibition of HAT indirectly affects DDR. Indeed, rtt109 mutants fail to reassemble chromatin after DSB and thus exhibit a checkpoint recovery defect [19]. Therefore, it is possible that curcumin inhibits DDR through its HAT inhibitor activity by way of a distinct mechanism that influences chromatin structure. We also noticed that high concentrations of VPA (10mM) are required to inhibit DDR. Because 50-fold less curcumin is required to suppress DDR, this agent may be a more effective sensitizer for DNA-damaging agents.

In conclusion, our findings indicate that curcumin increases the sensitivity of yeast to DNA damage by inhibiting DDR. Curcumin counteracts multiple DDR pathways, including the G2/M DNA damage checkpoint and homologous recombination (S5 Fig). After DSB induction, DNA resection proceeds normally, but the recruitment of Mec1-Ddc2 is inhibited by curcumin, which may in turn inhibit the activation of H2A and Rad53[60]. Furthermore, curcumin inhibits homologous recombination, presumably by promoting the degradation of Rad52, and this effect is dependent on its HDAC inhibitor activity. Consequently, cell death is promoted cooperatively through increased damage-induced apoptosis and the elimination of autophagy induced by DNA-damaging compounds.

## Supporting Information

S1 Fig Ddc2 protein levels are not affected by curcumin following DNA damage. *DDC2-MYC* (RLY001) cells were arrested in G2 and the HO endonuclease was induced by the addition of galactose to generate a DSB. The culture was split and treated with or without 200 μM curcumin. Samples were processed for western blotting using Myc antibodies. Amido black staining and Pgk1 protein serve as loading controls.
(TIF)

S2 Fig (A) Curcumin inhibits DSB repair in gcn5 mutants. PCR analysis of the WT (YMV045) and *gcn5∆* (DHY003) strains following induction of an HO lesion as in Fig 2B. (B) Curcumin promotes Rad52 degradation in gcn5 mutants. WT (YMV045) and *gcn5∆* (YAY017) strains were cultured as in Fig 3A and processed for western blotting using HA antibodies.
(TIF)

S3 Fig (A) Rad51 recombinase is inhibited by curcumin. As described in Fig 4A, we detected the total protein expression of Rad51. (B) The DNA damage response is counteracted by curcumin following MMS and 4NQO treatment. BY4741 cells or *RAD52-HA* (YAY014) cells were cultured as in Fig 3, and DNA damage was induced by MMS or 4NQO. Samples were processed for western blotting using the indicated antibodies.
(TIF)

S4 Fig Total HDAC protein levels are not affected by curcumin in response to a DSB. YMV045 cells were cultured as in Fig 3 and processed by western blotting using the indicated antibodies.
(TIF)

S5 Fig Model of the roles of HDAC inhibition in the DNA damage response. (TIF)

S1 Table Genotypes of yeast strains used in this study. (DOCX)
