# Supplementary figures and images for: Curcumin-Mediated HDAC Inhibition Suppresses the DNA Damage Response and Contributes to Increased DNA Damage Sensitivity
Source: PLoS One. 2015 Jul 28;10(7):e0134110. doi: 10.1371/journal.pone.0134110 (PMC4517890; doi:10.1371/journal.pone.0134110)

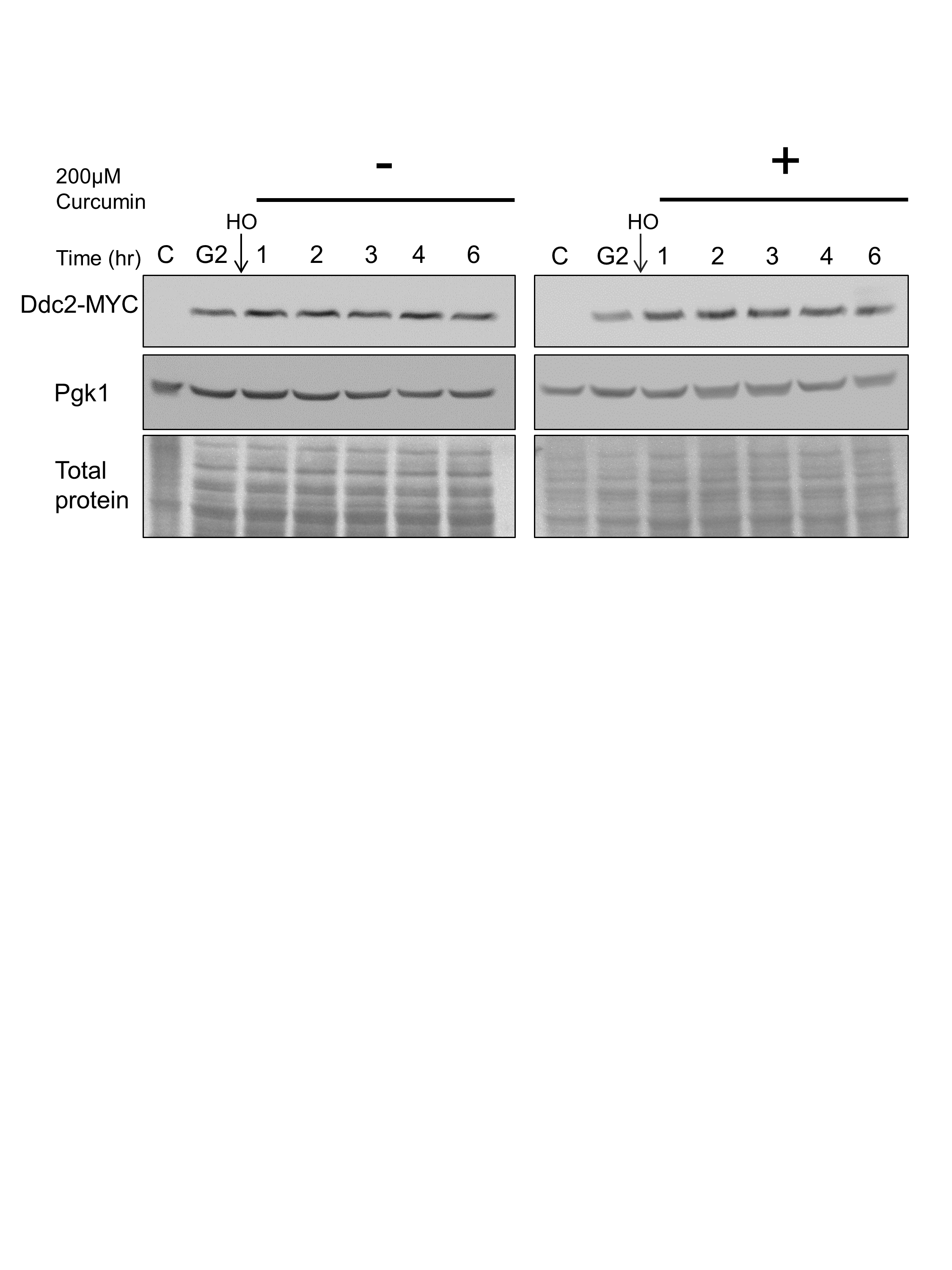

Supplement: S1 Fig — DDC2-MYC (RLY001) cells were arrested in G2 and the HO endonuclease was induced by the addition of galactose to generate a DSB. The culture was split and treated with or without 200 μM curcumin. Samples were processed for western blotting using Myc antibodies. Amido black staining and Pgk1 protein serve as loading controls. (TIF) [file pone.0134110.s001.TIF]

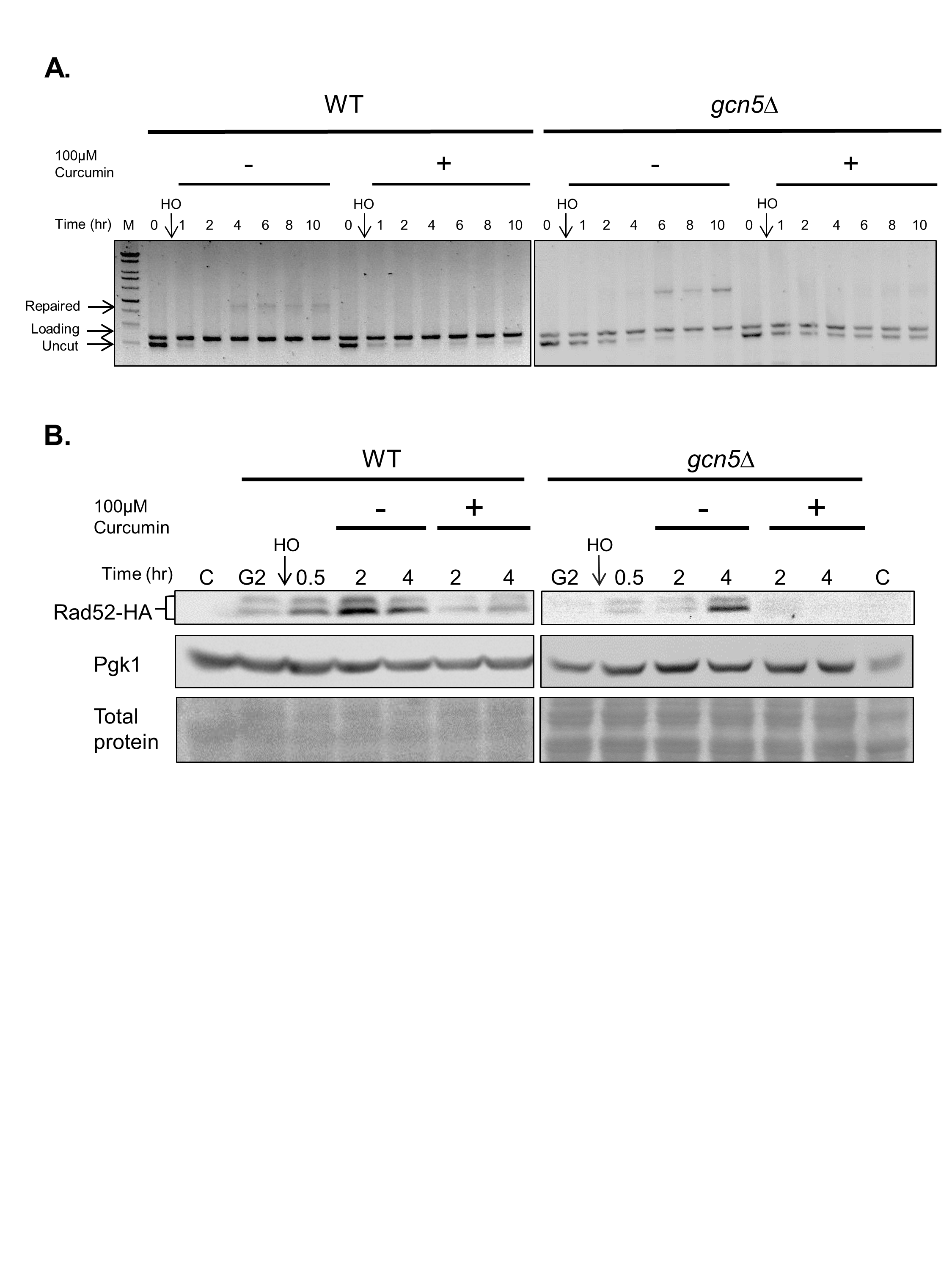

Supplement: S2 Fig — WT (YMV045) and gcn5∆ (YAY017) strains were cultured as in Fig 3A and processed for western blotting using HA antibodies. (TIF) [file pone.0134110.s002.TIF]

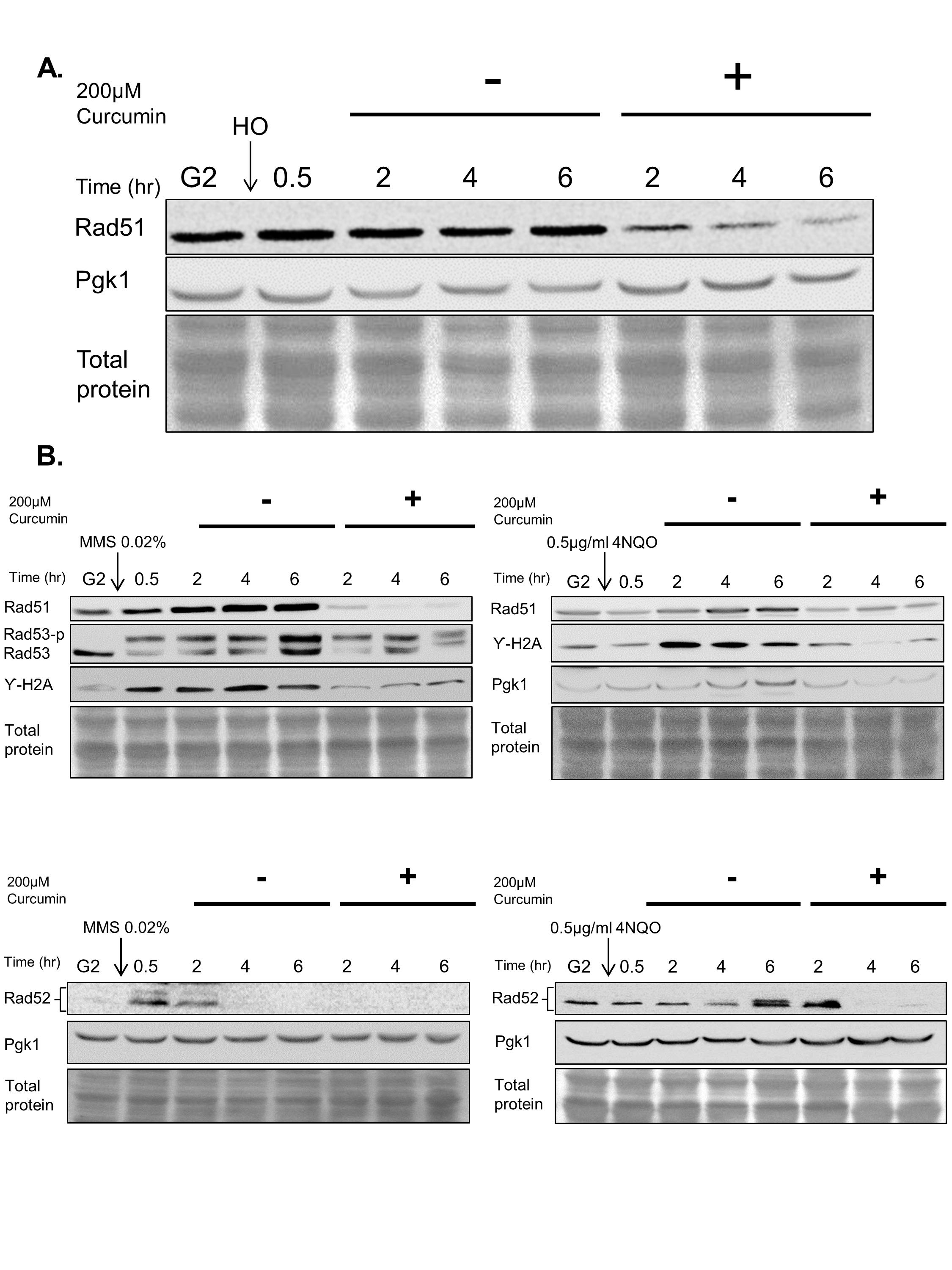

Supplement: S3 Fig — BY4741 cells or RAD52-HA (YAY014) cells were cultured as in Fig 3, and DNA damage was induced by MMS or 4NQO. Samples were processed for western blotting using the indicated antibodies. (TIF) [file pone.0134110.s003.TIF]

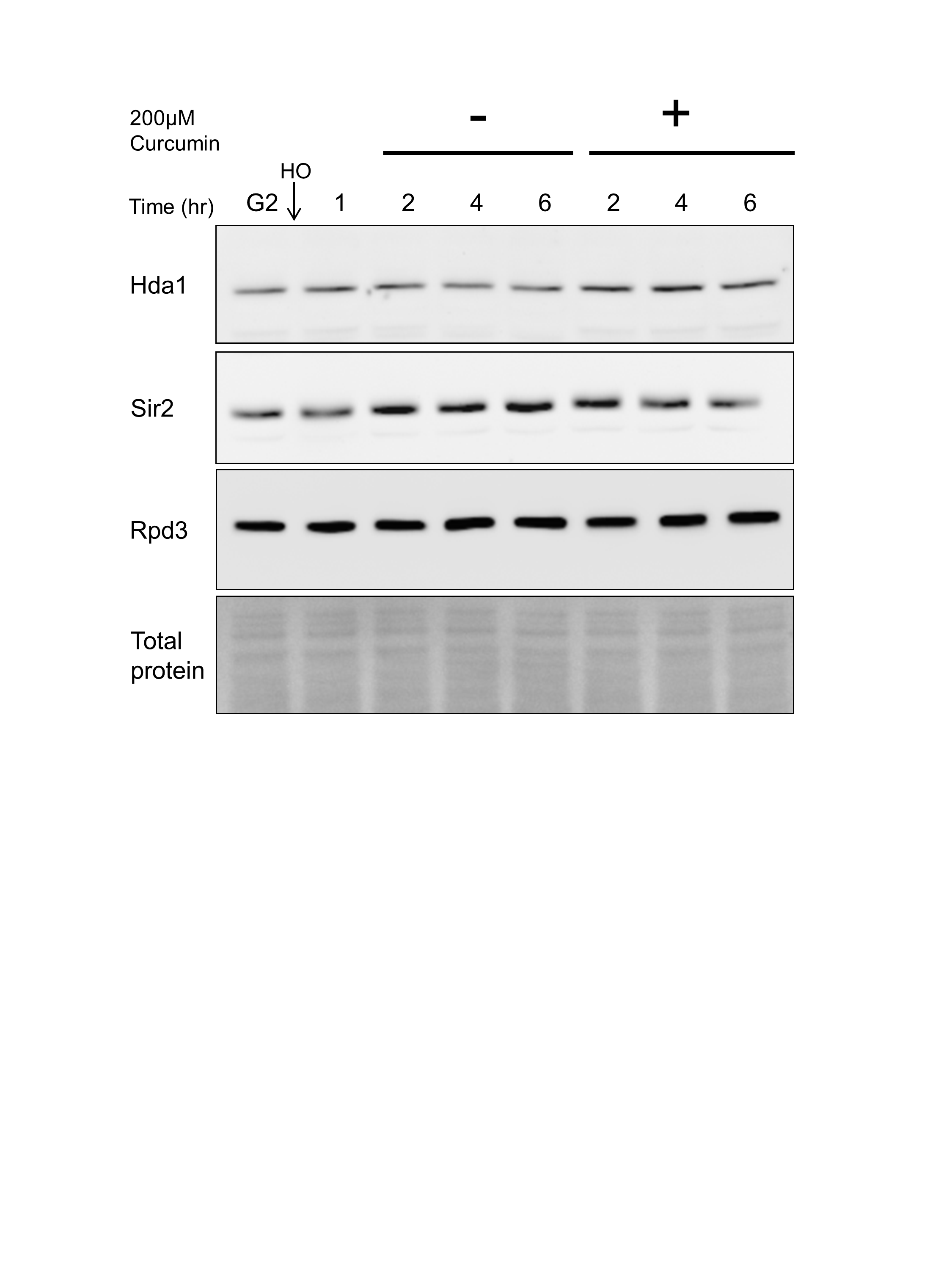

Supplement: S4 Fig — YMV045 cells were cultured as in Fig 3 and processed by western blotting using the indicated antibodies. (TIF) [file pone.0134110.s004.TIF]

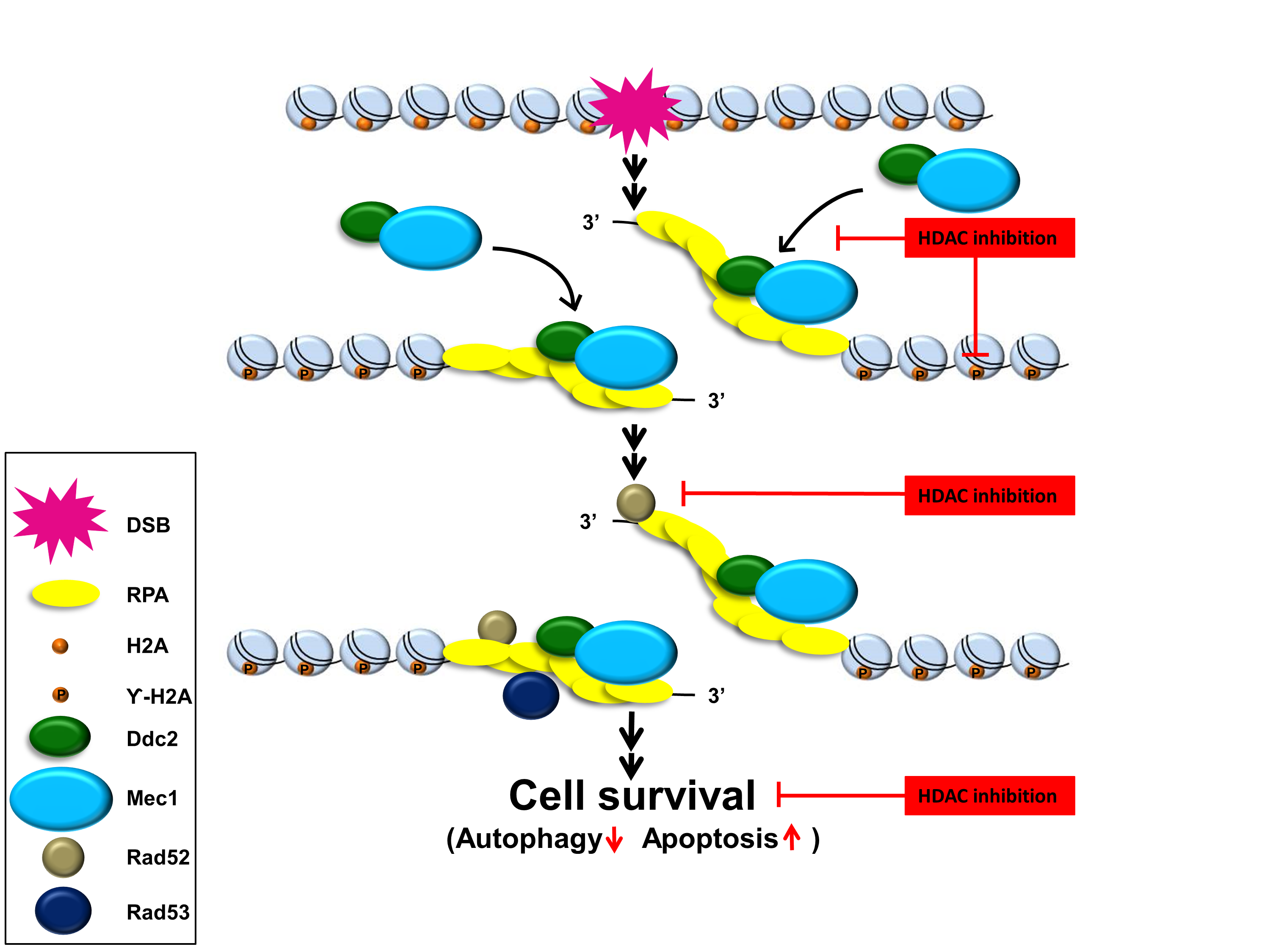

Supplement: S5 Fig — (TIF) [file pone.0134110.s005.TIF]
