# Supplementary material for: Curcumin-Mediated HDAC Inhibition Suppresses the DNA Damage Response and Contributes to Increased DNA Damage Sensitivity
Source: PLoS One. 2015 Jul 28;10(7):e0134110. doi: 10.1371/journal.pone.0134110 (PMC4517890; doi:10.1371/journal.pone.0134110)
Supplement: S1 Table — (DOCX) [file pone.0134110.s006.docx]

**S1 Table Genotypes of yeast strains used in this study.**

| Strain | Genotype | Source |
| --- | --- | --- |
| YMV002 | *MATα ho hml∆::ADE1 mata∆::ADE1 his4::-URA3-leu2-(Xho1 to Asp718)-his4 leu2::HOcs ade3::GAL::HO ade1 lys5 ura3-52* | James E. Haber |
| YMV037 | *MATα ho hml∆::ADE1 mata∆::ADE1 his4::-URA3-leu2-(Xho1 to Asp718)-his4 leu2::HOcs ade3::GAL::HO ade1 lys5 ura3-52 rad52∆::HPH* | James E. Haber |
| YMV045 | *MATα ho hml∆::ADE1 mata∆::hisG hmr∆::ade1 leu2::leu2 (Asp-718-SalI)-URA3-pBR322-HOcs ade3::GAL::HO ade1 lys5 ura3-52 trp1* | James E. Haber |
| YMV046 | *MATα ho hml∆::ADE1 mata∆::hisG hmr∆::ADE1 leu2:HOcs ade3::GAL::HO ade1 lys5 ura3-52 trp1 rad52∆::HPH* (hygro) | James E. Haber |
| YMV 057 | *MATα ho hml∆::ADE1 mata∆::hisG hmr∆::ade1 leu2::leu2 (Asp-718-SalI)-URA3-pBR322-HOcs ade3::GAL::HO ade1 lys5 ura3-52 trp1 srs2::HPH* | James E. Haber |
| BY4741 | *MATa his3∆ leu2∆ met15∆ ura3∆* | This study |
| BY4741-atg1 | *MATa his3∆ leu2∆ met15∆ ura3∆ atg1∆::KAN* | This study |
| BY4741-atg8 | *MATa his3∆ leu2∆ met15∆ ura3∆ atg8∆::KAN* | This study |
| BY4741-gcn5 | *MATa his3∆ leu2∆ met15∆ ura3∆ gcn5∆::KAN* | This study |
| BY4741-hat1 | *MATa his3∆ leu2∆ met15∆ ura3∆ hat1∆::KAN* | This study |
| BY4741-hda1 | *MATa his3∆ leu2∆ met15∆ ura3∆ hda1∆::KAN* | This study |
| BY4741-rad6 | *MATa his3∆ leu2∆ met15∆ ura3∆ rad6∆::KAN* | This study |
| BY4741-rpd3 | *MATa his3∆ leu2∆ met15∆ ura3∆ rpd3∆::KAN* | This study |
| BY4741-sir2 | *MATa his3∆ leu2∆ met15∆ ura3∆ sir2∆::KAN* | This study |
| BY4741-rtt109 | *MATa his3∆ leu2∆ met15∆ ura3∆ rtt109∆::KAN* | This study |
| CCY025 | *MATα ho hml::ADE1 mata::hisG hmr::ADE1 leu2 (Asp-718-SalI)-URA3-pBR322-HOcs ade3::GAL::HO ade1 lys5 ura3-52 DDC1-MYC::TRP* | Jessica Tyler |
| RLY001 | *MATα ho hml∆::ADE1 mata∆::hisG hmr∆::ade1 leu2::leu2 (Asp-718-SalI)-URA3-pBR322-HOcs ade3::GAL::HO ade1 lys5 ura3-52 trp1 (trp1::hisG) DDC2-MYC::KAN* | This study |
| DHY003 | *MATα ho hml∆::ADE1 mata∆::hisG hmr∆::ade1 leu2::leu2(Asp-718-SalI)-URA3-pBR322-HOcs ade3::GAL::HO ade1 lys5 ura3-52 trp1 (trp1::hisG) gcn5∆::KAN* | This study |
| YAY012 | *MATα ho hml∆::ADE1 mata∆::hisG hmr∆::ade1 leu2::leu2(Asp-718-SalI)-URA3-pBR322-HOcs ade3::GAL::HO ade1 lys5 ura3-52 trp1 (trp1::hisG) rpd3∆::KAN* | This study |
| YAY013 | *MATα ho hml∆::ADE1 mata∆::hisG hmr∆::ade1 leu2::leu2 (Asp-718-SalI)-URA3-pBR322-HOcs ade3::GAL::HO ade1 lys5 ura3-52 trp1 (trp1::hisG) HA-RAD52::KAN* | This study |
| YAY016 | *MATα ho hml∆::ADE1 mata∆::hisG hmr∆::ade1 leu2::leu2(Asp-718-SalI)-URA3-pBR322-HOcs ade3::GAL::HO ade1 lys5 ura3-52 trp1 (trp1::hisG) HA-RAD52::KAN rpd3∆::trp* | This study |
| YAY028 | *MATα ho hml∆::ADE1 mata∆::hisG hmr∆::ade1 leu2::leu2(Asp-718-SalI)-URA3-pBR322-HOcs ade3::GAL::HO ade1 lys5 ura3-52 trp1 (trp1::hisG) MYC-RAD52::TRP* | This study |
| YAY032 | *MATα ho hml∆::ADE1 mata∆::hisG hmr∆::ade1 leu2::leu2(Asp-718-SalI)-URA3-pBR322-HOcs ade3::GAL::HO ade1 lys5 ura3-52 trp1 (trp1::hisG?) Mre11- MYC::KAN* | This study |
| RLY004 | BY4741-atg8 tranformed with PRS416 GFP-Atg8 in URA drop media | This study |
| RLY005 | BY4741-atg1 tranformed with PRS416 GFP-Atg8 in URA drop media | This study |
| RLY006 | *MATαho hml∆::ADE1 mata∆::hisG hmr∆::ade1 leu2::leu2(Asp-718-SalI)-URA3-pBR322-HOcs ade3::GAL::HO ade1 lys5 ura3-52 trp1 (trp1::hisG) MYC-RAD52::TRP sem1::KAN* | This study |
| RLY007 | *MATα ho hml∆::ADE1 mata∆::hisG hmr∆::ade1 leu2::leu2(Asp-718-SalI)-URA3-pBR322-HOcs ade3::GAL::HO ade1 lys5 ura3-52 trp1 (trp1::hisG) MYC-RAD52::TRP hat1∆::KAN* | This study |
